# Body Segment Differences in Surface Area, Skin Temperature and 3D Displacement and the Estimation of Heat Balance during Locomotion in Hominins

**DOI:** 10.1371/journal.pone.0002464

**Published:** 2008-06-18

**Authors:** Alan Cross, Mark Collard, Andrew Nelson

**Affiliations:** 1 Laboratory of Human Evolutionary Studies, Department of Archaeology, Simon Fraser University, Burnaby, British Columbia, Canada; 2 Department of Anthropology, University of Western Ontario, London, Canada; University of Cambridge, United Kingdom

## Abstract

The conventional method of estimating heat balance during locomotion in humans and other hominins treats the body as an undifferentiated mass. This is problematic because the segments of the body differ with respect to several variables that can affect thermoregulation. Here, we report a study that investigated the impact on heat balance during locomotion of inter-segment differences in three of these variables: surface area, skin temperature and rate of movement. The approach adopted in the study was to generate heat balance estimates with the conventional method and then compare them with heat balance estimates generated with a method that takes into account inter-segment differences in surface area, skin temperature and rate of movement. We reasoned that, if the hypothesis that inter-segment differences in surface area, skin temperature and rate of movement affect heat balance during locomotion is correct, the estimates yielded by the two methods should be statistically significantly different. Anthropometric data were collected on seven adult male volunteers. The volunteers then walked on a treadmill at 1.2 m/s while 3D motion capture cameras recorded their movements. Next, the conventional and segmented methods were used to estimate the volunteers' heat balance while walking in four ambient temperatures. Lastly, the estimates produced with the two methods were compared with the paired t-test. The estimates of heat balance during locomotion yielded by the two methods are significantly different. Those yielded by the segmented method are significantly lower than those produced by the conventional method. Accordingly, the study supports the hypothesis that inter-segment differences in surface area, skin temperature and rate of movement impact heat balance during locomotion. This has important implications not only for current understanding of heat balance during locomotion in hominins but also for how future research on this topic should be approached.

## INTRODUCTION

Heat balance is a key variable in the assessment of the locomotor energetics of humans and other hominins [1]–[10]. Measured in Watts, heat balance is the difference between heat production and heat loss. As such, it is an indicator of how close to thermal equilibrium an individual is in a given ambient temperature. A positive value for heat balance means that an individual is producing and/or absorbing more heat than they can dissipate, while a negative value means that they are losing more heat than they can produce.

The conventional method for estimating a living human's heat balance during locomotion involves three steps. First, the focal individual's weight, height, walking/running speed and mean skin temperature while walking or running are recorded, along with the ambient temperature. Next, the following equations for heat production and heat loss are solved [1]–[4]:
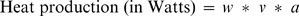
(1)

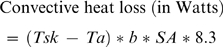
(2)

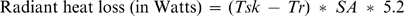
(3)

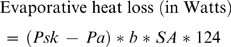
(4)The terms *w* and *v* in Equation 1 are the individual's weight in kilograms and velocity in meters per second, respectively. The term *a* in Equation 1 is a constant pertaining to the production of heat by metabolism and work. Normally *a* is assumed to equal 2 for walking and 4 for running [11]. The term *Ta* in Equation 2 is ambient temperature in degrees centigrade. The term *Tsk* in Equations 2 and 3 is mean skin temperature in degrees centigrade in *Ta*. The term *b* in Equations 2 and 4 is the square root of airflow over the skin in meters per second. The latter is usually taken to be equivalent to *v* on the grounds that a moving body creates its own wind. The term *SA* in the three equations is the total surface area of the skin in square centimeters. This value is usually estimated from the individual's height and weight with the aid of a regression equation presented by DuBois and DuBois [12]. According to these authors, surface area is given by the following equation:
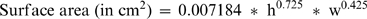
(5)where *h* is height in centimeters and *w* is weight in kilograms. The term *Tr* in Equation 3 is the radiant temperature. It is usually assumed to be equal to *Ta* (e.g. 2). The terms *Psk* and *Pa* in Equation 4 are the saturated water vapor pressure at skin temperature and the water vapor pressure of ambient air, respectively. The term 8.3 in Equation 2 is a heat transfer coefficient, as is the term 5.2 in Equation 3. The term 124 in Equation 4 is also a heat transfer coefficient. Lastly, a heat balance value for the individual is calculated by summing the estimates for convective and radiant heat loss, and then subtracting the resulting figure from the estimate for heat production. Methods of estimating heat balance during locomotion for extinct hominins proceed in a similar fashion [5]–[10]. The main difference is that estimated values are employed for all variables.

As recent work attests, the conventional method of assessing an individual's heat balance during locomotion is capable of yielding important insights [1]–[10]. Nevertheless, there are reasons to be skeptical about the estimates it produces. The most significant of these is the way it treats the body. Five of the eight variables included in the equations for heat production and heat loss—weight, speed, mean skin temperature, total skin surface area and rate of airflow over the skin—involve the individual whose heat balance is being estimated; the other variables—ambient temperature, the saturated water vapor pressure at skin temperature and the water vapor pressure of ambient air—are environmental. Weight, speed, mean skin temperature and total skin surface area all pertain to the body as a whole. Rate of airflow over the skin is also a whole-body variable. Given that rate of airflow is assumed to be the same as the individual's walking or running speed, the implicit assumption is that it acts equally on all parts of the body. Thus, the conventional method effectively treats the body as an undifferentiated mass. Dealing with the body in this manner is problematic because the segments of the body differ with respect to several variables that can affect thermoregulation. These include surface area, skin temperature, rate of movement, muscle mass, adipose tissue thickness, sweat gland response to increases in core body temperature and exposure to height-above-ground differences in ambient temperature [7], [13]. The impact of disregarding inter-segment differences is likely to be especially great when comparing early and later hominins, as body proportions change markedly during the course of human evolution [14]–[15]. Smaller but nonetheless significant differences in body proportions have been documented among regional populations of living humans [16]. Hence, the impact of disregarding inter-segment differences is also likely to be pronounced on comparisons of living humans from different regions of the world.

In this paper we report a study that investigated the impact on heat balance during locomotion of inter-segment differences in three variables: surface area, skin temperature and rate of movement. The approach adopted in the study was to generate heat balance estimates with the conventional method, and then compare those estimates with heat balance estimates obtained with a method that takes into account inter-segment differences in surface area, skin temperature and rate of movement (hereinafter, the ‘segmented method’). The rationale for this course of action was that, if the hypothesis that inter-segment differences in surface area, skin temperature and rate of movement affect heat balance during locomotion is correct, the estimates yielded by the conventional method and the segmented method should be statistically significantly different.

**Figure 1:**
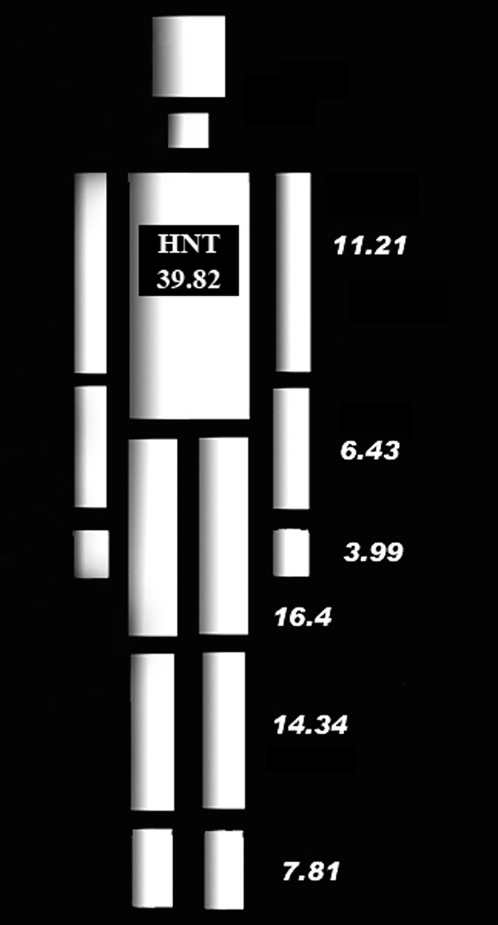
Model of the human body used in the segmented method. Numeric values represent the mean percentages of total body surface area represented by the various body segments based on the sample employed in the study reported here. Each segment is modeled as a cylinder. HNT  =  head, neck and trunk.

In the study heat balance was calculated as the difference between heat production and the sum of convective and radiant heat loss. Evaporative heat loss was omitted because the laboratory was not equipped to measure the saturated water vapor pressure at skin temperature or the water vapor pressure of ambient air, and evaporative heat loss data of the type needed for use in the segmented method could not be acquired from the literature. Apart from not employing the equation for evaporative heat loss, the conventional method was implemented as described earlier. The segmented method was developed specifically for the study reported here. It contrasts with the conventional method in that it treats the body as a collection of cylinders (Figure 1) that differ not only in their sizes but also in their movements during locomotion. Generating a heat balance estimate with the segmented method involves five steps. First, a range of whole-body and segment-specific anthropometric variables are recorded on an individual. Next, with a view to estimating segment-specific wind speeds, the individual's movements while walking or running on a treadmill are recorded with the aid of three-dimensional (3D) motion capture equipment. Thereafter, the surface areas and displacement rates of the individual's body segments are estimated. The surface area of a segment is calculated from its length and the mean of its proximal, middle and distal circumferences using the formula for determining the surface area of a cylinder. The displacement rate of a segment is calculated from the 3D motion data, and is equal to the vector sum of the distance the segment moves in the X, Y and Z planes in the course of a cycle divided by the duration of the cycle (in seconds). A cycle is delimited by two consecutive heel strikes of the dominant foot. Subsequently, these data are combined with walking speed, ambient temperature and segment-specific skin temperatures to solve the equation for heat production outlined in the previous section (Equation 1) and the following equations for convective heat loss and radiant heat loss:
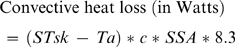
(6)

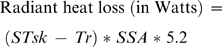
(7)where *Ta* is ambient temperature in degrees centigrade; *Tr* is radiant temperature; *STsk* is segment specific skin temperature in degrees centigrade in *Ta*; *c* is the segment specific displacement rate in meters per second; *SSA* is the segment surface area in square centimeters; and 8.3 and 5.2 are heat transfer coefficients. The last step in the segmented method is to sum the estimates for convective heat loss and radiant heat loss, and then subtract the resulting figure from the estimate for heat production. The resulting value is the individuals' heat balance.

## METHODS

Seven students from the University of Western Ontario in London, Ontario, participated in the study. The students were male and were aged between 23 and 26. Six of the volunteers were Euro-Canadians. The remaining volunteer was born in East Africa. None of the volunteers engaged in athletic activities on a regular basis, but they all reported being in good physical condition. The Ethics Review Board of the University of Western Ontario approved the study (Review #11120E), and the volunteers provided written informed consent.

First, 45 anthropometric variables were recorded on the seven volunteers. The variables recorded were stature, weight and the length and upper, middle and lower circumferences of the head and neck, trunk, upper arms, lower arms, hands, upper legs, lower legs and feet. The measurements were defined as per Gordon et al. [17]. Stature and weight were measured with an anthropometer and a standard analog scale, respectively. The lengths and circumferences of the body segments were measured with a steel measuring tape. Stature and the other linear measurements were recorded in centimeters; weight was recorded in kilograms. AC collected the anthropometric data.

Next, the movements of the volunteers were recorded in 3D while they walked on a treadmill wearing reflective markers. The motion capture facility is housed in the National Research Council's Virtual Environment Technologies Centre, which is part of its Integrated Manufacturing Technology Institute, located in London, Ontario. Ambient temperature in the laboratory was 22°C. All the volunteers walked barefoot on the treadmill wearing a form-fitting shirt and a pair of cycling shorts. The treadmill was a standard fitness industry model. The markers were placed at the proximal and distal borders of the volunteers' body segments. Where two segments articulate with each other, a single marker was used for the proximal border of one segment and the distal border of the other (e.g. the elbow marker was used for both the distal upper arm and the proximal lower arm). Markers were attached in various ways. The markers for the sternum and limbs were attached directly to the skin using two-sided tape, while those for the waist and hips were attached to a custom-made elastic belt. The head marker was attached to a form-fitting hat. The 3D recording equipment comprised eight Falcon HR240 cameras, and a Motion Integrated Data Acquisition System (MIDAS) computer running the program EVaRT 4.3. Two cameras were located on each side of the room ∼3 m above the floor. Prior to marker placement, the volunteers were given two minutes to become accustomed to walking on the treadmill. Once the markers were in place, they were asked to walk for a further five minutes in order to get used to wearing the markers. They were then asked to walk at 1.2 m/s. They were allowed to walk for two minutes before data recording commenced in order to ensure that they had acquired a normal gait. Data recording continued for three cycles. Sub-sampling rate was set at 60 Hz, 480 lines. All data were recorded in real time, were calibrated in millimetres and are accurate to within 0.8 mm. Motion capture data were recorded only once for each individual. AC also collected the motion capture data.

The 3D motion capture data for one volunteer (V5) were found to be unusable as a result of technical difficulties. Consequently, this individual was dropped from the sample.

Subsequently, each of the remaining volunteers' heat balance while walking was estimated with the conventional method and segmented method. The two methods of estimating heat balance were implemented as described in the last paragraph of the Introduction. All estimates were calculated as if the individuals were naked. Four estimates were generated with the conventional method. The first was calculated with ambient temperature (*Ta*) set at 20°C and the second with *Ta* set at 25°C. The third estimate was calculated with *Ta* set at 30°C and the fourth with *Ta* set 35°C. In line with previously published studies (e.g. 2), *Tr* values were assumed to be equal to values for *Ta*, and velocity (*v*) was set at walking speed, 1.2 m/s. Also in line with previously published studies, the constant pertaining to the production of heat by metabolism and work while walking (*a*) was assumed to be 2 and the square root of airflow over the skin in meters per second (*b*) was assumed to be the same as *v*. Since the laboratory was not equipped to measure skin temperature, mean skin temperature (*Tsk*) for each *Ta* was derived from the values presented by Houdas and Ring [18]. To minimize inaccuracy, a weighted mean *Tsk* was employed. Each segment *Tsk* was weighted according to the percentage of total surface area it represents (Figure 1) and then the average of the segment *Tsk* values was calculated. Four estimates were also generated with the segmented method using the same *Ta* values as were used to generate the conventional method estimates. Again, in all four calculations, *Tr* was assumed to be equal to *Ta* and (*a*) was set at 2. Total segment displacement was calculated by averaging the vector sums of 3D displacement of the proximal and distal segment markers over three cycles. The displacement rate of each segment (*c*) was computed by dividing the average total segment displacement per cycle (in meters) by the duration of that cycle (in seconds) while walking at 1.2 m/s. Once again, segment skin temperatures (*STsk*) were derived from the segment-specific skin temperature values presented by Houdas and Ring [18]. Total body convective heat loss and total body radiant heat loss were estimated by summing the segment specific heat loss values.

Lastly, the heat balance estimates produced with the conventional method and the segmented method were compared statistically with paired t-tests (p = 0.05). To reiterate, the expectation was that, if the hypothesis is correct and inter-segment differences in surface area, skin temperature and rate of movement affect heat balance, the heat balance estimates yielded by the conventional and segmented methods should be statistically significantly different. This analysis was carried out with the aid of SPSS 11 for Mac OS X.

## RESULTS

Table 1 presents the total surface area estimates produced by the conventional method and the segmented method. The mean total surface area estimate produced by the conventional model is 19,834 cm^2^. The mean total surface area estimate produced by the segmented method is 19,344 cm^2^. A paired t-test of the total surface area estimates generated by the two methods indicates that they are not significantly different (p = 0.74).

**Table 1:** Total surface areas obtained with the conventional and segmented methods.

Volunteer	Conventional method	Segmented method
V1	20898.0	20098.4
V2	18882.9	17913.4
V3	17624.4	16753.2
V4	20400.3	19927.5
V6	19442.1	19165.7
V7	21753.7	22207.9
Mean	19833.6 (SD 1488.5)	19344.4 (SD 1892.2)

All values in cm^3^.

Table 2 presents the estimated surface areas of the segments into which the body is divided in the segmented method. As anticipated, the surface areas of the individual body segments vary considerably. In addition, there is conspicuous inter-individual variation in the scale of the differences among segments. In one individual (V4) the surface area of the largest segment (the trunk) is 23 times larger than the smallest segment (the neck). In the other individuals, the differences between the largest and smallest segment surface areas are smaller but still considerable, the largest surface areas being between 13 and 14 times larger than the smallest surface areas. It is also evident that there is inter-individual variation in the relative size of the segments. In all six individuals, the neck has the smallest surface area and the hands have the next smallest surface area. The relative sizes of the feet, upper arms and trunk are also consistent across the sample. In all six individuals, the feet are the fifth largest segment, the upper arms the sixth and the trunk the ninth. However, the relative sizes of the lower arms, upper legs and lower legs vary among the individuals. For example, in subject V2 the lower arms are ranked fourth, the lower legs seventh and the upper legs eighth, while in subject V3 the lower arms, lower legs and upper legs are ranked third, eighth and seventh, respectively. Thus, the segment specific estimated surface areas support the notion that the segments of the body vary markedly in parameters that can impact thermoregulation.

**Table 2:** Total and segment-specific surface areas for the study sample.

Volunteer	TSA	Head	Neck	Trunk	UA	LA	Hands	UL	LL	Feet
V1	20098.4	1241.2	503.1	6802.7	2175.6	1258.6	816.0	2945.0	2968.6	1387.6
V2	17913.4	1118.7	475.8	6239.1	1918.0	1086.4	824.2	2716.8	2229.0	1305.4
V3	16753.2	1235.2	238.5	5603.3	1770.0	966.2	691.8	2507.8	2555.0	1185.4
V4	19927.5	1271.6	523.3	7163.8	1955.6	1107.4	860.2	3057.6	2692.8	1295.2
V6	19165.7	1133.6	470.8	6368.9	1934.2	1167.8	869.4	2989.0	2824.8	1407.2
V7	22207.9	1062.1	513.3	6417.3	2223.4	1438.6	996.0	4245.8	3740.4	1571.0

TSA  =  total surface area. UA  =  upper arms. LA  =  lower arms. UL  =  upper legs. LL  =  lower legs. All values in cm^3^.

**Figure 2:**
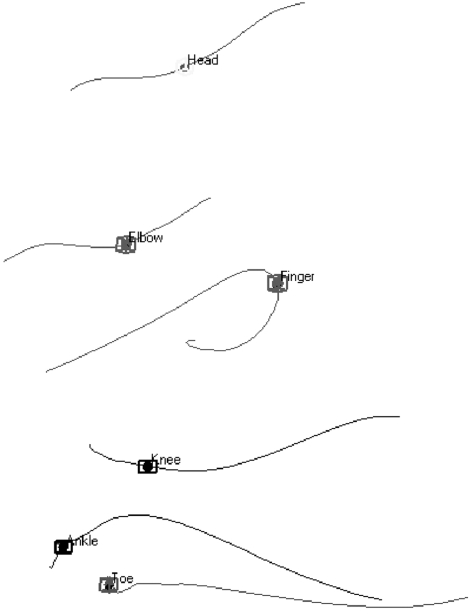
2D rendering of 3D movement of selected markers during a one-second period of normal walking.

The motion capture data indicate that each body segment follows a distinct displacement pattern during normal walking (Figure 2). Consequently, each segment traverses a different amount of space per cycle and possesses a different rate of displacement (Table 3). When the segments are ranked according to their amount of displacement per cycle, those of the upper limb traverse the most space. During normal walking, the segments of the upper limb have both a forward and backward swing while the segments of the lower limb have only a forward swing followed by a stationary phase where the body pivots above the foot in contact with the substrate. As a result, the lower arms traverse 25% more space than the trunk at a rate 50% faster than walking speed, and the hands traverse 46.5% more space than the trunk at a rate 93% faster than walking speed. Each individual swung one arm more than the other with bilateral differences in hand displacement ranging from 4%-29%. Bilateral displacement differences are not seen in the lower limb. Interestingly, arm swing asymmetry is not correlated with handedness. Inter-individual variation in displacement patterns was limited in the head, neck and trunk segment and the lower limb segments, but marked in the segments of the upper limbs, particularly the lower arms and hands. Given that the additional swinging of the upper limbs can be expected to result in greater wind exposure for the upper limb segments in each walking cycle, the motion capture data also support the notion that the segments of the body vary markedly in parameters that impact thermoregulation, and argue against the use of a single value for wind speed.

**Table 3:** Mean segment displacement distances and rates per cycle.

Segment	Displacement distance	Displacement rate
Hands	241.86 (SD 54.94)	2.16 (SD 0.39)
Lower arms	202.56 (SD 34.16)	1.81 (SD 0.22)
Upper arms	169.53 (SD 17.38)	1.51 (SD 0.07)
Upper legs	167.34 (SD 10.58)	1.49 (SD 0.03)
Lower legs	166.09 (SD 8.80)	1.48 (SD 0.04)
Feet	164.13 (SD 7.69)	1.47 (SD 0.06)
Trunk	155.54 (SD 13.14)	1.39 (SD 0.03)
Head and neck	154.75 (SD 14.09)	1.38 (SD 0.04)

Displacement distances in cm. Displacement rates in m/sec.

Table 4 presents the mean convective and radiant heat loss estimates for the 14 body segments (values for left and right sides pooled) when the ambient temperature was set at 20°C, 25°C, 30°C and 35°C, together with the percentage of total heat loss dissipated by each segment. As anticipated, there is considerable variation among the segments' heat loss. For example, at 20°C the head/neck/trunk segment is responsible for nearly 60% of convective and radiant heat loss. The legs dissipate approximately 25% of the heat produced while the arms dissipate only about 18%.

**Table 4:** Mean total and segment-specific heat loss.

Temperature	THL	HNT	Arm	Leg	UA	LA	Hand	UL	LL	Foot
20°C	253.07 (SD 23.82)	56.05 (SD 3.46)	18.01 (SD 0.91)	25.97 (SD 2.68)	9.73 (SD 0.33)	5.97 (SD 0.54)	2.31 (SD 0.26)	14.66 (SD 1.76)	9.93 (SD 1.09)	1.39 (SD 0.07)
25°C	175.65 (SD 16.38)	56.82 (SD 3.38)	16.41 (SD 0.82)	26.77 (SD 2.73)	10.16 (SD 0.34)	5.92 (SD 0.54)	0.34 (SD 0.03)	14.71 (SD 1.82)	9.59 (SD 1.06)	2.47 (SD 0.13)
30°C	114.95 (SD 10.94)	48.41 (SD 3.24)	18.93 (SD 0.87)	32.66 (SD 2.48)	9.10 (SD 0.28)	6.14 (SD 0.51)	3.69 (SD 0.39)	13.88 (SD 1.59)	10.14 (SD 1.07)	8.64 (SD 0.41)
35°C	17.70 (SD 1.76)	45.11 (SD 3.04)	35.46 (SD 1.73)	19.43 (SD 1.47)	19.14 (SD 0.60)	8.88 (SD 0.83)	7.45 (SD 0.80)	2.65 (SD 0.32)	9.77 (SD 1.12)	7.01 (SD 0.37)

THL  =  total heat loss. HNT  =  head, neck and trunk segments. UA  =  upper arm segments. LA  =  lower arm segments. UL  =  upper leg segments. LL  =  lower leg segments. Total heat loss values in Watts. Segment heat loss values presented as percentages of total body heat loss.

Table 5 gives the average surface areas, displacement rates, skin temperatures and heat loss values for the various body segments in the four ambient temperatures. It is clear from these figures that displacement rate affects the heat balance estimates yielded by the segmented method. A comparison of the heat loss of the hands and forearms at 35°C illustrates this. At 35°C, the hands contribute nearly the same amount to total heat loss as the forearms (1.32 Watts versus 1.57 Watts) even though their surface area is 62% of the surface area of the forearms, and their skin temperature is only 0.1°C higher than that of the forearms. The figures presented in Table 5 also show that surface area affects the heat balance estimates yielded by the segmented method. For example, when ambient temperature is 20°C, the upper leg contributes 59% more to total heat loss than the lower arm even though they have comparable skin surface temperatures (27.9 Watts and 27.7 Watts, respectively) and the lower arm moves 21% more per cycle than the upper leg. Thus, in this case, surface area clearly has a greater impact on heat loss than segment displacement rate. Lastly, the figures presented in Table 5 show that skin temperature affects the heat balance estimates yielded by the segmented method. Specifically, the closer the skin temperature of a segment is to ambient temperature, the smaller the segment's contribution to total heat loss no matter how great its surface area or displacement. Conversely, the greater the difference between skin segment temperature and ambient temperature, the greater the impact of displacement. The hand exemplifies this. When the ambient temperature is 25°C, the skin temperature of the hand is only 0.4°C above the ambient temperature and it loses less than 1 watt. In contrast, when the ambient temperature is 20°C, the skin temperature of the hand is 4°C above the ambient temperature and it loses nearly 6 Watts. Thus, the heat balance estimates yielded by the segmented method are the consequence of the interplay of segment specific surface areas, skin temperatures *and* displacement rates.

**Table 5:** Mean segment surface areas, displacement distances, skin temperatures and heat loss.

Segment	SA	DD	Tsk20	HL20	Tsk25	HL25	Tsk30	HL30	Tsk35	HL35
Upper arms	1996.13	169.53	28.0	24.62	30.8	17.85	33.4	10.46	36.1	3.39
Lower arms	1170.83	202.56	27.7	15.11	30.3	10.40	33.6	7.06	35.8	1.57
Hands	842.93	241.46	24.0	5.85	25.4	0.60	32.9	4.24	35.9	1.32
Upper legs	3077.00	167.34	27.9	37.10	30.5	25.84	33.4	15.96	35.1	0.47
Lower legs	2835.1	166.09	25.8	25.13	28.9	16.84	32.7	11.66	35.4	1.73
Feet	1358.63	164.13	21.7	3.52	27.1	4.34	34.8	9.93	35.6	1.24
Head and neck	1631.2	154.75	32.9	141.85	33.9	99.80	34.8	55.65	35.9	7.98
Trunk	6432.52	155.54	31.3	141.85	33	99.80	34.5	55.65	35.6	7.98

SA  =  surface area. DD  =  displacement distance during one cycle. Tsk20  =  skin temperature when ambient temperature is 20°C. HL20  =  heat loss at 20°C. Tsk25  =  skin temperature when ambient temperature is 25°C. HL25  =  heat loss at 25°C. Tsk30  =  skin temperature when ambient temperature is 30°C. HL25  =  heat loss at 30°C. Tsk35  =  skin temperature when ambient temperature is 35°C. HL35  =  heat loss at 35°C. Surface areas in cm^3^. Displacement distances in cm. Skin temperatures in °C. Heat loss values in Watts.

The estimates of heat production, heat loss and heat balance produced with the conventional and segmented methods are given in Tables 6, 7, 8, 9. The methods produce similar general patterns with respect to the impact of ambient temperature on heat balance. There is no overlap among the four sets of heat balance estimates produced with the conventional method or among the four sets of estimates produced with the segmented method. In each case, all the heat balance estimates obtained when the ambient temperature was set at 20°C are lower than those obtained when the ambient temperature was set at 25°C, and the latter are all lower than the heat balance estimates obtained when the ambient temperature was set at 30°C. Likewise, all the heat balance estimates obtained when the ambient temperature was set at 30°C are lower than those obtained when the ambient temperature was set at 35°C. In addition, both methods indicate that, for the sample employed, the optimal temperature in which to walk is between 20°C and 25°C. Both methods yielded negative heat balance estimates at 20°C, and positive heat balance estimates at 25°C, 30°C and 35°C. Furthermore, both methods indicate that radiant and convective heat loss is ineffective for dissipating heat when the ambient temperature is 35°C. When *Ta* was set at 35°C, the conventional method suggested that the average heat loss would equal approximately 8% of heat production, while the segmented method suggested that it would equal approximately 9% of heat production.

**Table 6:** Estimates of heat production, convective heat loss, radiant heat loss and heat balance using the conventional method and the segmented method when *Ta* is 20°C.

	Conventional method				Segmented method			
Volunteer	HP	C	R	HB	HP	C	R	HB
V1	213.6	153.87	88.0	−28.27	213.6	177.72	92.46	−56.58
V2	178.8	137.14	78.43	−36.77	178.8	155.97	80.43	−57.60
V3	164.4	128.26	73.35	−37.21	164.4	144.19	74.54	−54.33
V4	194.4	152.56	87.45	−45.61	194.4	173.44	90.31	−69.35
V6	186.0	146.73	83.92	−44.65	186.0	160.83	85.25	−60.08
V7	199.7	170.02	97.23	−67.55	199.7	188.35	95.42	−84.07
Mean	189.4	148.10	84.73	−43.34	189.4	166.75	86.57	−63.67

HP  =  heat production. C  =  convective heat loss. R  =  radiant heat loss. HB  =  heat balance. All values in Watts.

**Table 7:** Estimates of heat production, convective heat loss, radiant heat loss and heat balance using the conventional method and the segmented method when *Ta* is 25°C.

	Conventional method				Segmented method			
Volunteer	HP	C	R	HB	HP	C	R	HB
V1	213.6	107.45	61.45	44.70	213.6	123.58	64.44	25.58
V2	178.8	95.77	54.77	28.26	178.8	108.13	55.99	14.68
V3	164.4	89.57	51.22	23.61	164.4	100.19	51.91	12.30
V4	194.4	106.54	60.93	26.93	194.4	120.19	62.85	11.36
V6	186.0	102.46	58.60	24.94	186.0	111.50	58.97	15.53
V7	199.7	118.73	67.90	13.07	199.7	129.89	66.25	3.56
Mean	189.4	103.42	59.15	26.92	189.4	115.58	60.07	13.84

HP  =  heat production. C  =  convective heat loss. R  =  radiant heat loss. HB  =  heat balance. All values in Watts.

**Table 8:** Estimates of heat production, convective heat loss, radiant heat loss and heat balance using the conventional method and the segmented method when *Ta* is 30°C.

	Conventional method				Segmented method			
Volunteer	HP	C	R	HB	HP	C	R	HB
V1	213.6	65.42	37.42	110.76	213.6	80.15	41.57	91.88
V2	178.8	58.31	33.35	87.14	178.8	71.16	36.43	71.21
V3	164.4	54.53	31.19	78.68	164.4	65.59	33.75	65.06
V4	194.4	64.86	37.10	92.44	194.4	78.25	40.49	75.66
V6	186.0	62.38	35.68	87.94	186.0	73.51	38.58	73.91
V7	199.7	72.29	41.34	86.07	199.7	86.6	43.60	69.50
Mean	189.4	62.97	36.01	90.05	189.4	75.88	39.07	74.54

HP  =  heat production. C  =  convective heat loss. R  =  radiant heat loss. HB  =  heat balance. All values in Watts.

**Table 9:** Estimates of heat production, convective heat loss, radiant heat loss and heat balance using the conventional method and the segmented method when *Ta* is 35°C.

	Conventional method				Segmented method			
Volunteer	HP	C	R	HB	HP	C	R	HB
V1	213.6	10.60	6.06	196.94	213.6	12.55	6.47	194.58
V2	178.8	9.45	5.40	163.95	178.8	11.15	5.65	162.00
V3	164.4	8.83	5.05	150.52	164.4	10.23	4.69	149.48
V4	194.4	10.51	6.01	177.88	194.4	12.09	6.18	176.13
V6	186.0	10.11	5.78	170.11	186.0	11.37	5.94	168.69
V7	199.7	11.71	6.70	181.29	199.7	13.32	6.55	179.83
Mean	189.4	10.20	5.83	173.45	189.4	11.79	5.91	171.79

HP  =  heat production. C  =  convective heat loss. R  =  radiant heat loss. HB  =  heat balance. All values in Watts.

While the two methods yield similar general patterns with respect to the impact of ambient temperature on heat balance, the estimates of heat balance produced with the segmented method are consistently lower than those obtained with the conventional method. When the ambient temperature was 20°C, the mean segmented estimate was 20.63 Watts lower than the mean conventional estimate (−63.97 Watts versus −43.34 Watts). When the ambient temperature was 25°C, the mean segmented estimate was 13.08 Watts lower than the mean conventional estimate (13.84 Watts versus 26.92 Watts). When the ambient temperature was 30°C, the mean segmented estimate was 15.63 Watts lower than the mean conventional estimate (74.54 Watts versus 90.17 Watts). When the ambient temperature was 35°C, the mean segmented estimate was 1.66 Watts lower than the mean conventional estimate (171.79 Watts versus 173.45 Watts). According to the paired t-tests, all of the differences between the estimates yielded by the two methods are highly significant (p = 0.000). Thus, the segmented method yields significantly lower estimates of heat balance during walking than the conventional method when ambient temperature is between 20°C and 35°C.

## DISCUSSION

The results of the comparison of the heat balance estimates yielded by the conventional and segmented methods are consistent with the predictions of the hypothesis that inter-segment differences in surface area, skin temperature and rate of movement impact heat balance during locomotion. Potentially, this has important implications for research on heat balance during locomotion in living humans and extinct hominins. Needless to say, however, for this to be the case the results in question need to be reliable.

To date, we have identified, or have had brought to our attention, four aspects of our study that have the potential to affect the reliability of its results. The first is the small size of the sample. While we would have liked to incorporate more individuals in the study, the sample is not unusually small when compared to those used in the studies that prompted our research. For example, the regression equation that is used to estimate surface area in the conventional method was derived from a sample of eight individuals [12]. Nevertheless, it is possible that the results of the analyses would have been different with a larger sample. To assess this possibility, we compared the mean statures and weights of our volunteers to those recorded by Gordon et al. [17] on a sample of 1774 male U.S. Army personnel. We also compared the mean total surface areas of the two samples as determined by the segmented method. The mean stature of our six volunteers was 179.85 cm (SD = 10.92). Their mean weight was 78.92 kg (SD = 7.11). The mean total surface area of our volunteers was 19,344 cm^2^ (SD = 1,892). Gordon et al.'s [17] sample possessed a mean stature of 175.58 cm (SD = 6.68), a mean weight of 78.49 kg (SD = 11.10) and a mean total surface area of 19,809 cm^2^. The close similarity between the stature, weight and total surface area means of the two samples suggests that our sample is a reasonable representation of the variability among males living in North America.

The second aspect of our study that has the potential to affect the reliability of its results is the use of published skin temperatures. Although it is not uncommon for estimated values to be used in heat balance studies (e.g. 1–2), the use of published data undoubtedly reduced the accuracy of our results. However, there is no reason to think that directly measuring segment-specific skin temperature would have affected our central finding—that the conventional method yields significantly higher heat balance estimates than the segmented method. This is because the same skin temperature values were used with both the conventional method and the segmented method. Incorporating directly measured skin temperature values might have affected the differences in heat balance among individuals but it is unlikely that it would have eliminated the difference between the two sets of heat balance estimates.

The third aspect of our study that has the potential to affect the reliability of its results is the omission of evaporative heat loss. To reiterate, we did not estimate evaporative heat loss because the laboratory was not equipped to measure the saturated water vapor pressure at skin temperature or the water vapor pressure of ambient air, and evaporative heat loss data of the type needed for use in the segmented method could not be acquired from the literature. Again, there is no reason to think that including evaporative heat loss would have eliminated the difference between the two sets of estimates. Recently, Buono [19] has shown that the segments of the human body differ substantially in terms of sweat gland response to changes in core body temperature during exercise. For example, the number of active sweat glands in the forearm increased by approximately 600% as core body temperature rose from 37.4°C to 38.3°C, while over the same temperature range the number of active sweat glands in the back increased by less than 100%. This suggests that the segments of the human body likely differ in terms of evaporative heat loss. In our view, it is implausible that factoring in another variable that differs among segments would have led to a reduction in the difference between the results yielded by the conventional and segmented methods. Rather, it is likely that incorporating evaporative heat loss would have resulted in an even greater difference between the heat balance estimates yielded by the two methods.

The fourth aspect of our study that has the potential to affect the reliability of its results is the use of cylinders to represent all the segments of the body in the segmented method. Intuitively, it seems likely that it would have been better to model some segments of the body as frustums (truncated cones) rather than as cylinders, and that our failure to do so may have contributed to the segmented method yielding significantly lower heat balance estimates than the conventional method. To evaluate this possibility, we recalculated the estimates of total surface area and convective heat loss at 30°C for one of the volunteers, V1, using the formula for the surface area of a frustum to calculate the surface areas of the segments that are usually closer in shape to truncated cones than to cylinders (upper arms, lower arms, upper legs and feet) and the formula for the surface area of a cylinder to calculate the surface areas of the remaining segments. The surface area estimate generated when the upper arms, lower arms, upper legs and feet were modeled as frustums was 6.3 cm^2^ lower than the surface area estimate generated when they were modeled as cylinders. This equates to a difference of only 0.03%. The estimates of convective heat loss were also very close. When V1's upper arms, lower arms, upper legs and feet were modeled as frustums his convective heat loss at 30°C was estimated to be 0.05 Watts or 0.06% lower than when these segments were modeled as cylinders. Given that the difference between the two sets of estimates for V1 is negligible, and that the convective heat loss estimate obtained when the upper arms, lower arms, upper legs and feet were modeled as frustums is *lower* than the one obtained when they were modeled as cylinders, it is unlikely that the significant difference between the heat balance estimates yielded by the conventional and segmented methods is an artifact of the use of cylinders to represent all the segments of the body in the segmented method.

It appears, then, that the results of the study are reliable. There is no reason to think that the difference between the heat balance estimates yielded by the conventional and segmented methods would have been eliminated if a larger sample had been employed or if skin temperature had been directly measured. There is also no reason to think that the difference between the heat balance estimates yielded by the conventional and segmented methods would have been eliminated if evaporative heat loss had been taken into account or if the segments of the body had been modeled as a combination of frustums and cylinders rather than just as a collection of cylinders. Accordingly, it seems reasonable to conclude that inter-segment differences in surface area, skin temperature and rate of movement do indeed impact heat balance across a wide range of the ambient temperatures experienced by living humans and extinct hominins.

The study's support for the hypothesis that inter-segment differences in surface area, skin temperature and rate of movement impact heat balance casts doubt on the results obtained with the conventional method of estimating heat balance during locomotion in previous studies (e.g. 1–10). Specifically, since the heat balance estimates yielded by the segmented method are significantly lower than the heat balance estimates yielded by the conventional method, it is likely that heat balance during locomotion has been overestimated repeatedly. Accordingly, there is a need for studies in which the hypotheses that have been tested with the conventional method are retested with a segmented method of estimating heat balance during locomotion. Given that the impact of disregarding inter-segment differences is likely to be especially pronounced when comparing early and late hominin species or humans from different regions of the world, revisiting the conclusions of studies that have carried out such comparisons (e.g. 4, 7) should be a particular priority. The study's support for the hypothesis that inter-segment differences in surface area, skin temperature and rate of movement impact heat balance also suggests that any new hypothesis regarding heat balance during locomotion in hominins that is developed should be tested from the outset with a segmented method rather than the conventional method. Lastly, as we noted earlier, surface area, skin temperature and rate of movement are only three of the variables that both differ among the segments of the human body and have the potential to impact thermoregulation. Other variables that fall into this category include muscle mass, adipose tissue thickness, sweat gland response to rises in core temperature, and exposure to height-above-ground differences in ambient temperature. The results of the study suggest the impact on heat balance of inter-segment differences in these additional variables should also be investigated.
